# Secondary matrix‐associated autologous chondrocyte implantation after failed cartilage repair shows superior results when combined with autologous bone grafting: Findings from the German Cartilage Registry (KnorpelRegister DGOU)

**DOI:** 10.1002/ksa.12467

**Published:** 2024-09-15

**Authors:** Johannes Weishorn, Philipp Niemeyer, Peter Angele, Gunther Spahn, Thomas Tischer, Tobias Renkawitz, Yannic Bangert

**Affiliations:** ^1^ Department of Orthopaedics, Heidelberg University Hospital Ruprecht‐Karls‐University Heidelberg Heidelberg Germany; ^2^ OCM Orthopedic Surgery Munich Munich Germany; ^3^ Clinic for Orthopedics and Trauma Surgery Albert‐Ludwigs‐University Freiburg Freiburg im Breisgau Germany; ^4^ University Medical Center Regensburg Regensburg Germany; ^5^ Sporthopaedicum Regensburg/Straubing Regensburg Germany; ^6^ Center of Trauma and Orthopaedic Surgery at Jena University Hospital Jena Germany; ^7^ Malteser Waldkrankenhaus St. Marien Erlangen Germany; ^8^ Department of Orthopaecdics University Medical Center Rostock Rostock Germany

**Keywords:** cartilage, clinical study, knee, registry, treatment failure

## Abstract

**Purpose:**

The aim of this study was to evaluate whether additive autologous bone grafting (ABG) improves clinical outcome and survival in revision matrix‐associated autologous chondrocyte implantation (M‐ACI) after failed cartilage repair (CR).

**Methods:**

A retrospective, registry‐based, matched‐pair analysis was performed to compare patient‐reported outcomes and survival in secondary M‐ACI with or without additional bone grafting for focal full‐thickness cartilage defects of the knee and to compare it with those in primary M‐ACI. Patients were matched for age, sex, body mass index, defect size and localization, and number of previous CRs. The Knee Injury and Osteoarthritis Outcome Score (KOOS) was assessed over a follow‐up period of 36 months. The patient acceptable symptomatic state, the clinical response rate and the survival of the subgroups were determined.

**Results:**

A total of 818 patients were matched. Revision M‐ACI (*n* = 238) with concomitant bone grafting was associated with significantly higher PRO as measured by KOOS (80.8 ± 16.8 vs. 72.0 ± 17.5, *p* = 0.032) and higher CRR (81.4% vs. 52.0%, *p* = 0.018) at 36 months compared to patients with revision M‐ACI alone. KOOS and KOOS improvement in these patients did not differ from those who underwent primary M‐ACI (*p* = n.s.). The combination of M‐ACI and ABG resulted in a significantly higher KOOS at 36 months than M‐ACI alone, regardless of whether bone marrow stimulation (89.6 ± 12.5 vs. 68.1 ± 17.9, *p* = 0.003) or ACI (82.6 ± 17.0 vs. 72.8 ± 16.0, *p* = 0.021) was performed before. Additional bone grafting results in equivalent survival rates at 7 years in secondary compared to primary M‐ACI (83% vs. 84%, *p* = n.s.).

**Conclusions:**

Regardless of the type of previous CR, additional bone grafting in secondary M‐ACI improves the clinical outcome, response rate and survival at 36 months compared to M‐ACI alone. Secondary M‐ACI with ABG had comparable clinical response and survival rates to primary M‐ACI. Therefore, subchondral bone should be treated even in cases of mild bone involvement in revision M‐ACI.

**Level of evidence:**

Level III.

**Abbreviations**
(M‐)ACI: (matrix‐associated) autologous chondrocyte implantation
ABG: autologous bone grafting
ADL: activities of daily living
BMI: body mass index
CR: cartilage repair
FT: femorotibial
FU: follow‐up
ICRS: International Cartilage Regeneration and Joint Preservation Society
KOOS: Knee Injury and Osteoarthritis Outcome Score
MCID: minimal clinically important difference
PASS: patient acceptable symptomatic state
PF: patellofemoral
PRO(M): patient reported outcome (measure)
PSM: propensity score matching
QOL: quality of life

## INTRODUCTION

Matrix‐associated autologous chondrocyte implantation (MACI) is a widely used cartilage regeneration technique due to its regenerative potential and good long‐term clinical results [7, 30]. The current guideline of the DGOU “Clinical Tissue Regeneration Working Group” describes M‐ACI as first‐line therapy for defects larger than 2 cm^2^ [18]. Several studies have shown that previous failure of bone marrow stimulation (BMS) is a negative prognostic factor for secondary M‐ACI [14, 17, 23]. This ultimately led the UK National Institute for Health and Care Excellence to refuse reimbursement for M‐ACI in revision cases and recommend it as the best available first‐line treatment for cartilage defects >2 cm^2^ [15].

The osteochondral unit is thought to play an important role in articular CR and is the subject of intense research [16, 22, 26]. Subchondral bone involvement has been suggested to be associated with worse pain and symptoms after M‐ACI [11]. However, a recent analysis by Seiferth et al. showed that any failed cartilage repair (CR) at the defect site is a negative prognostic factor up to 3 years after M‐ACI, regardless of the type of previous treatment [25]. In particular, dysfunction of the osteochondral unit has been controversially discussed as a possible cause for the worse outcome of M‐ACI in CR revision [25, 29].

In a recently published study, secondary osteochondral allografts (OCAs) were shown to have similar clinical outcomes and survival compared to primary OCA [9]. The authors suggested the use of OCA and sandwich M‐ACI in secondary CR, although evidence for sandwich M‐ACI is still lacking. The combination of autologous bone grafting (ABG) and M‐ACI has been shown to be a reliable option for treating the osteochondral unit in primary CR in a recently published registry study [29]. Patients who underwent additional ABG had equivalent outcomes to those who underwent primary M‐ACI alone despite having more preoperative pain and worse joint function [29]. However, there is no evidence that combined M‐ACI and ABG is effective for revision CR in a representative cohort. It remains unclear whether the combination of M‐ACI and ABG can achieve reliable results in revision cases and whether these results are comparable to those after primary M‐ACI.

The aim of this registry‐based analysis was to address this gap by evaluating the effect of ABG in secondary M‐ACI, hypothesizing that patients would show differences in patient‐reported outcomes (PROMs) at 36 months.

## MATERIALS AND METHODS

The present study examines data from the German Cartilage Registry (KnorpelRegister DGOU) with the approval of the Ethics Commission of the Medical Center, University of Freiburg: EK‐FR 105/13_130795. As previously reported, the registry is a multicentre, longitudinal database of patients undergoing CR procedures [27, 29]. The registry is registered at germanctr.de (DRKS00005617) and is conducted in accordance with the Declaration of Helsinki. Written informed consent was obtained prior to cartilage therapy and patients were followed up by e‐mail to complete PRO questionnaires.

**Figure 1:**
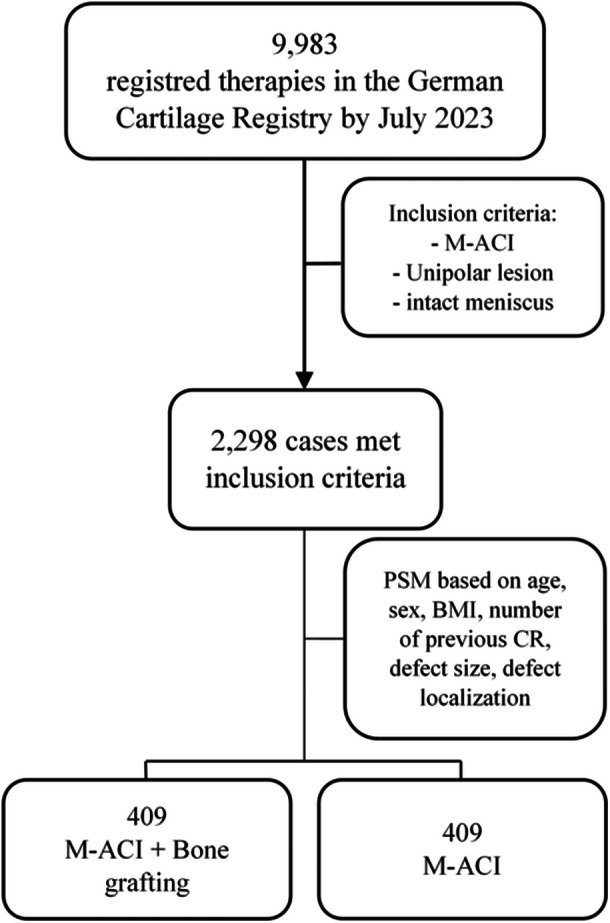
Flowchart visualizing patient selection and matching. BMI, body mass index; CR, cartilage repair; M‐ACI, (matrix‐associated) autologous chondrocyte implantation; PSM, propensity score matching.

All patients with a minimum age of 18 years at the time of surgery were screened for eligibility. Patients with unipolar, full‐thickness cartilage lesions treated with M‐ACI with or without concomitant bone grafting and an intact meniscus were included (Figure 1). Patients with bipolar defects, those who had concomitant procedures such as ligament reconstruction or realignment surgery, patients with deficient meniscus and those with moderate/severe osteoarthritis (Kellgren–Lawrence > 2) were excluded. Follow‐up (FU) was analysed at 6, 12, 24 and 36 months. Kaplan–Meier analysis was used to estimate survival up to 7 years. Baseline patient characteristics including body mass index (BMI), gender, age, number of previous CRs, defect size and defect site were collected.

### Outcome measures

Two treatment groups were created, M‐ACI with and without concomitant bone grafting, and patients were further subdivided into those with and without previous CR. The primary outcome measure was the Knee Injury and Osteoarthritis Outcome Score (KOOS) and its subscores at various FUs. As a secondary outcome, the change in KOOS (ΔKOOS) was calculated and evaluated within the treatment groups with respect to previous CR [4]. Patient acceptable symptomatic state (PASS) and minimal clinically important difference (MCID) were used to evaluate KOOS and ΔKOOS, respectively [5, 20]. As recently published, the MCID for overall KOOS was defined as an improvement of ≥10 from baseline [2, 28]. The clinical response rate (CRR), defined as the proportion of patients achieving the MCID, was also determined and compared between treatment groups and subgroups [4, 29]. Failure rates and estimated survival were analysed. Failure was defined as any reoperation on the defect side within the FU period.

### Sample size calculation

Based on expert opinion and previous studies, an a priori sample size calculation was performed assuming an expected difference in KOOS of 10 with an SD of 15 [2, 8, 20, 29]. To achieve the intended statistical power of 80% with an *α* of 0.05, at least 64 patients per group were needed.

### Matching

A 1:1 propensity score matching (PSM) with replacement was performed to identify groups with similar baseline characteristics and to reduce potential confounders. PSM was performed for baseline age, sex, number of previous CR procedures on the same defect, defect location and defect size. A matching tolerance of 0.01 was set to obtain groups with similar baseline characteristics. Two groups of 409 subjects with similar baseline characteristics were identified.

### Statistical analysis

Categorical variables are reported as numbers and percentages, and continuous variables are shown as means (SD). An unpaired *t* test for parametric distribution and the Mann–Whitney test for skewed data were used to analyse continuous variables. The *χ* ^2^ test was used to compare categorical variables between groups. The significance level was set at 0.05. Kaplan–Meier survival analysis was used to estimate the mean time to failure in different groups. Statistics were performed using SPSS version 29.0 (IBM) and G‐Power 3.1 (Heinrich Heine University).

## RESULTS

A total of 409 patients with similar baseline characteristics were identified in each treatment group (Table 1). Baseline demographic characteristics did not differ significantly between the two groups.

**Table 1:** Baseline demographic characteristics of M‐ACI patients with or without concomitant ABG (*n* = 818).

		M‐ACI			M‐ACI + ABG			*p*
No. of patients		409			409			
Age, years		30.4 (9.7)			29.9 (10.4)			n.s.
Sex								
m		261			257			n.s.
f		148			152			
BMI (kg/m^2^)		25.4 (4.1)			25.2 (3.9)			n.s.
Defect size, cm^2^		4.4 (2.2)			4.4 (2.3)			n.s.
Localization, %		**PF**	**FT**		**PF**	**FT**		n.s.
		83	326		73	336		
ICRS grade, *n*		**IV**			**IV**			n.s.
		409			409			
No. of previous CR, *n*		**0**	**1**	**≥2**	**0**	**1**	**≥2**	n.s.
		290	107	12	290	103	16	

*Note*: Mean (SD).

Abbreviations: ABG, autologous bone grafting; BMI, body mass index; CR, cartilage repair; f, female; FT, femorotibial; m, male; M‐ACI, matrix‐associated autologous chondrocyte implantation; n.s., not significant; PF, patellofemoral.

### Clinical results in primary and secondary M‐ACI

**Figure 2:**
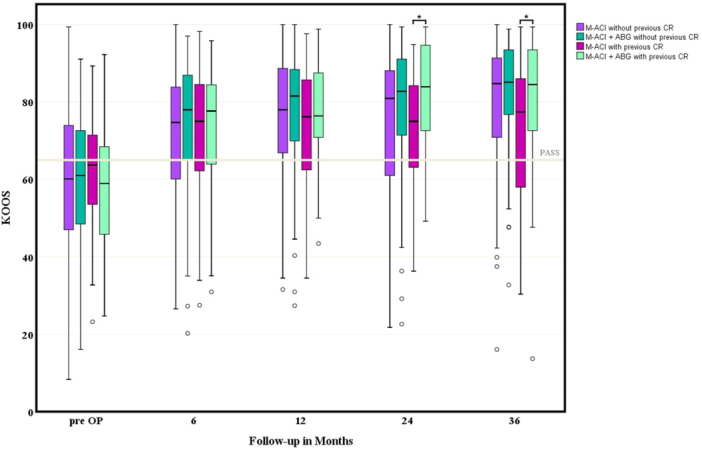
Mean Knee Injury and Osteoarthritis Outcome Score (KOOS) scores at different follow‐ups in the respective groups. ABG, autologous bone grafting; CR, cartilage repair; M‐ACI, matrix‐associated autologous chondrocyte implantation.

Patients with additional ABG for M‐ACI had a comparable preoperative clinical status as measured by KOOS at baseline (59.0 ± 16.8 vs. 62.2 ± 14.6 and 59.7 ± 15.8 vs. 61.1 ± 18.5, *p* = not significant [n.s.]) compared to patients with single M‐ACI treatment. KOOS at baseline was similar in patients with and without previous CR (*p* = n.s.). In patients with previous CR, PRO improved during the postoperative course, favouring patients with additional ABG (Figure 2).

Specifically, patients with secondary M‐ACI had significantly better KOOS at 24 months (*p* = 0.005) and 36 months (*p* = 0.032), and were significantly more likely to achieve PASS (*p* < 0.05) when treated with additional bone grafting. In patients without prior cartilage therapy, the difference in KOOS showed a similar trend, but the difference between the two groups did not reach significance (Table 2).

**Table 2:** Mean KOOS scores and the PASS rate at the different FUs in the respective groups.

	Patients, secondary M‐ACI (*n* = 238)			Patients, primary M‐ACI (*n* = 580)		
KOOS	M‐ACI	M‐ACI + ABG	*p*	M‐ACI	M‐ACI + ABG	*p*
Baseline	62.2 (14.6)	59.0 (16.8)	n.s.	61.1 (18.5)	59.7 (15.8)	n.s.
6 Months	71.0 (17.0)	73.0 (15.8)	n.s.	72.2 (15.1)	74.0 (16.6)	n.s.
% reach PASS	73.2	73.2	n.s.	67.4	74.1	n.s.
12 Months	72.7 (16.3)	76.4 (13.0)	n.s.	75.4 (16.5)	78.3 (15.3)	n.s.
% reach PASS	70.5	78.3	n.s.	77.0	80.6	n.s.
24 Months	72.5 (15.5)	81.9 (13.3)	**0.005** a	74.2 (18.7)	79.4 (15.5)	n.s.
% reach PASS	70.3	87.8	**0.034** a	70.2	84.3	**0.034** a
36 Months	72.0 (17.5)	80.8 (16.8)	**0.032** a	78.7 (18.2)	82.8 (13.4)	n.s.
% reach PASS	65.7	85.0	**0.047** a	80.4	91.8	**0.048** a

*Note*: Mean (SD).

Abbreviations: ABG, autologous bone grafting; KOOS, Knee Injury and Osteoarthritis Outcome Score; M‐ACI, matrix‐associated autologous chondrocyte implantation; n.s., not significant; PASS, patient acceptable symptomatic state.

a indicates significance.

Both groups, those receiving M‐ACI and those receiving combined treatment, showed clinically relevant improvement from baseline regardless of whether they had previously undergone CR or not (Table 3). In cartilage revision surgery, patients who received combined M‐ACI and ABG showed significantly greater improvements in total KOOS at 12, 24 and 36 months and achieved a significantly higher CRR at 36 months (*p* = 0.018). Slightly greater improvements in KOOS were also seen in patients with M‐ACI and ABG without prior CR. However, the difference between the two groups was not significant (*p* = n.s.).

**Table 3:** Mean ΔKOOS scores and the CRR at the different FUs in the respective groups.

	Secondary M‐ACI patients (*n* = 238)			Primary M‐ACI patients (*n* = 580)		
ΔKOOS	M‐ACI	M‐ACI + ABG	*p*	M‐ACI	M‐ACI + ABG	*p*
6 Months	9.5 (16.6)	14.2 (19.4)	n.s.	12.1 (18.6)	14.3 (13.9)	n.s.
CRR in %	52.4	62.5	n.s.	50.7	61.6	n.s.
12 Months	10.5 (16.1)	17.4 (18.2)	**0.036** a	15.4 (21.7)	18.6 (14.4)	n.s.
CRR in %	61.8	66.7	n.s.	61.7	68.7	n.s.
24 Months	10.2 (16.7)	22.9 (18.8)	**0.023** a	14.1 (21.7)	19.7 (16.1)	n.s.
CRR in %	60.7	69.0	n.s.	65.1	75.2	n.s.
36 Months	9.8 (18.8)	21.9 (17.7)	**0.027** a	18.6 (19.5)	23.1 (16.3)	n.s.
CRR in %	52.0	81.4	**0.018** a	70.0	82.9	n.s.

*Note*: Mean (SD).

Abbreviations: ABG, autologous bone grafting; CRR, clinical response rate; FUs, follow‐ups; KOOS, Knee Injury and Osteoarthritis Outcome Score; M‐ACI, matrix‐associated autologous chondrocyte implantation; n.s., not significant.

a indicates significance.

**Figure 3:**
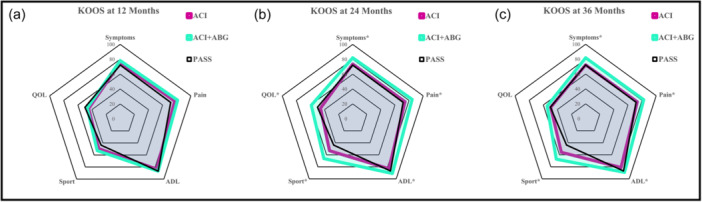
Differences in Knee Injury and Osteoarthritis Outcome Score (KOOS) subscores in secondary matrix‐associated autologous chondrocyte implantation (M‐ACI) between the two subgroups at 12 (a), 24 (b) and 36 months (c). *indicates significance. ABG, autologous bone grafting; ADL, activities of daily living; PASS, patient acceptable symptomatic state; QOL, quality of life.

A more detailed analysis in the subgroup of patients with secondary M‐ACI showed that patients treated with M‐ACI and ABG had significantly fewer symptoms (82.1 ± 14.9 vs. 73.6 ± 13.7 at 24 months and 81.9 ± 15.9 vs. 72.4 ± 16.0 at 36 months) and pain scores (84.4 ± 14.4 vs. 74.3 ± 17.4 at 24 months and 82.4 ± 18.5 vs. 73.2 ± 20.0 at 36 months). They had significantly higher KOOS‐Sport (66.3 ± 26.2 vs. 53.2 ± 27.9 at 24 months and 67.3 ± 27.2 vs. 55.8 ± 27.0 at 36 months) and activities of daily living (91.1 ± 10.4 vs. 83.1 ± 14.8 at 24 months and 89.2 ± 15.9 vs. 81.1 ± 17.7 at 36 months) scores compared to patients treated with ACI alone in revision CR (Figure 3). Patients treated with M‐ACI combined with ABG also showed a better quality of life at 24 months (58.8 ± 22.5 vs. 45.8 ± 21.9; *p* = 0.012).

Differences in KOOS were additionally analysed for patients according to the type of previous CR (Table 4). Patients with ABG had significantly higher KOOS at 36 months irrespective of whether BMS (89.6 ± 12.5 vs. 68.1 ± 17.9) or ACI (82.6 ± 17.0 vs. 72.8 ± 16.0) was performed prior to revision M‐ACI. Regardless of the type of prior CR, revision M‐ACI with additional bone grafting showed comparable outcomes to primary M‐ACI (89.6 ± 12.5 vs. 82.8 ± 13.4 and 82.6 ± 17.0 vs. 82.8 ± 13.4, *p* = n.s.).

**Table 4:** KOOS analysis in secondary M‐ACI with prior CR.

	Secondary M‐ACI with prior BMS			Secondary M‐ACI with prior ACI		
KOOS	M‐ACI	M‐ACI + ABG	*p*	M‐ACI	M‐ACI + ABG	*p*
Baseline	56.3 (16.4)	59.5 (16.9)	n.s.	62.9 (13.7)	56.7 (16.6)	n.s.
6 Months	71.2 (19.0)	74.9 (19.1)	n.s.	70.3 (16.7)	72.6 (15.8)	n.s.
12 Months	75.7 (13.1)	75.5 (15.2)	n.s.	71.1 (17.5)	76.5 (13.1)	n.s.
24 Months	75.0 (13.8)	85.9 (13.9)	0.023^a^	71.4 (16.8)	80.5 (13.3)	0.024^a^
36 Months	68.1 (17.9)	89.6 (12.5)	0.003^a^	72.8 (16.0)	82.6 (17.0)	0.021^a^

*Note*: *p* values.

Abbreviations: ABG, autologous bone grafting; ADL, activities of daily living; BMS, bone marrow stimulation; CR, cartilage repair; KOOS, Knee Injury and Osteoarthritis Outcome Score; M‐ACI, matrix‐associated autologous chondrocyte implantation; QOL, quality of life.

a indicates significance.

### Reoperation rate and time to reoperation

**Figure 4:**
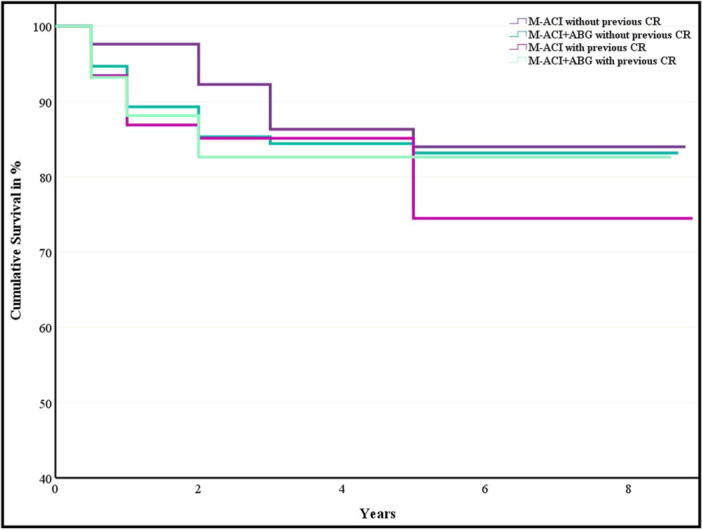
Kaplan–Meier plot illustrating estimated survival over time. ABG, autologous bone grafting; CR, cartilage repair; M‐ACI, matrix‐associated autologous chondrocyte implantation.

A total of 55 reoperations were performed in the present cohort. Estimated survival to the endpoint of reoperation for any reason was 84% (SD: 4.6) at 7.8 years for isolated M‐ACI and 83% (SD: 3.4) at 7.5 years for M‐ACI and ABG in primary CR. For revision M‐ACI, the estimated survival was 74% (SD: 6.4) at 7.3 years for isolated M‐ACI and 83% (SD: 5.0) at 7.3 years for combined M‐ACI and ABG (*p* = n.s., *χ* ^2^ = 0.2; Figure 4).

## DISCUSSION

The main finding of the present analysis was that in secondary M‐ACI surgery, a higher KOOS (80.8 ± 16.8 vs. 72.0 (17.5); *p* = 0.032) and a higher treatment response rate (81.4% vs. 52.0%, *p* = 0.018) were observed at 36 months with the combination of M‐ACI and ABG compared to M‐ACI alone. The combination of M‐ACI and ABG showed comparable response rates of 82.9% and 81.4% at 36 months after primary and secondary M‐ACI, respectively. In contrast, secondary M‐ACI alone showed an inferior CRR (52.0%). The observed benefits of concomitant treatment of the subchondral bone in revision M‐ACI were independent of whether BMS or ACI had been previously performed. Compared to primary M‐ACI, the addition of ABG results in equivalent survival rates in secondary M‐ACI.

M‐ACI is a well‐studied procedure for treating even large chondral defects in the knee, achieving reliable, improved clinical results and having a positive effect on potential osteoarthritis progression [3, 6, 7, 12]. However, several studies have reported inferior M‐ACI outcomes in patients with prior BMS [14, 17, 23, 24]. The inferiority of microfracture and microdrilling compared to M‐ACI has been demonstrated in several studies and therefore represents an inferior treatment option according to current evidence, especially for large defects [1, 10, 19]. It has been hypothesized that the cartilage may be more susceptible to damage and further degeneration due to the BMS and the subsequent thickening of the subchondral bone with a corresponding reduction in the thickness of the overlying cartilage [14]. Also, the poorer tissue quality of the regenerated cartilage after BMS and the degree of defect filling have been discussed in this context [18]. Therefore, previous failed BMS treatment has been considered a reason for poorer outcomes in secondary M‐ACI [14]. It has been theorized that this altered and stiff subchondral plate leads to poorer results after M‐ACI in revision cases [14].

Interestingly, a large registry‐based analysis recently showed that outcome in secondary M‐ACI is also worse with previous cell‐based therapies and this compromise is not limited to BMS as the primary CR procedure [25]. Accordingly, the outcome in revision M‐ACI is compromised by any previous CR procedure, whereas previous knee surgery without CR seems to have no negative effect on the outcome after M‐ACI [25]. It is interesting to note that in osteochondral lesions and osteochondrosis dissecans, a combined treatment with M‐ACI and additional bone grafting can lead to comparable clinical results and survival rates as the primary treatment of pure chondral defects [29, 31]. In a comparative analysis, Merkely et al. [13] showed that severe bone marrow oedema is a significant predictor of graft failure in patients treated with secondary M‐ACI. The present analysis could show that favourable clinical outcomes and survival rates, similar to those in primary M‐ACI, can be achieved with the addition of ABG in secondary M‐ACI, regardless of the type of previous CR procedure. In contrast, the previously reported inferior outcomes in secondary M‐ACI without addressing the subchondral bone could be confirmed.

This suggests that a dysfunctional osteochondral unit may be a major contributor to the failure of M‐ACI surgery, which is supported by the findings of Gracitelli et al. [9] who analysed secondary OCAs and found similar results to primary OCA. Therefore, they suggested the use of OCA or sandwich M‐ACI in secondary cartilage surgery. However, evidence on the efficacy of sandwich M‐ACI in secondary cartilage surgery was lacking. The present analysis was able to show that secondary M‐ACI with concomitant ABG as a sandwich technique can provide comparable clinical results to primary M‐ACI at 36 months. Patients treated with secondary M‐ACI alone have inferior clinical outcomes and significantly lower CRRs at 36 months. Furthermore, Ogura et al. [21] showed inferior survival rates in secondary M‐ACI (62% vs. 81% at 5 years) compared to patients without previous CR. The present evaluation showed that estimated survival in secondary M‐ACI is comparable to that in primary M‐ACI (83% vs. 84%, n.s.) at 7 years when additional ABG is performed in revision CR. This is in line with the results published by Gracitelli et al. [9] who reported an 82% survival rate at 10 years after OCA in secondary cartilage surgery.

In summary, this study was the first to show that combined M‐ACI and ABG treatment can provide high postoperative survival and PRO in revision CR, comparable to that seen in primary M‐ACI. The results of the present study suggest that additional treatment of the subchondral bone should be considered in secondary M‐ACI, even in cases where the subchondral bone does not appear to be primarily involved. However, further research is needed to identify patient‐specific and radiographic predictors in patients who may benefit from additional subchondral bone treatment without having an indication for combined treatment based on current recommendations. Individualized treatment based on improved patient selection may lead to favourable outcomes in revision M‐ACI.

Despite the large cohort size and clinical relevance of the present study, several limitations must be considered when interpreting the data. Due to the retrospective nature of the registry data analysis, no definitive conclusions can be drawn regarding the observed differences in PRO between sandwich M‐ACI and M‐ACI alone in revision cartilage surgery. Unfortunately, magnetic resonance imaging data were not available and PRO could only be compared for the 3‐year period due to the limited power at later FUs. Excluding concomitant therapies allows for better comparability as differences in outcomes are limited to M‐ACI and the effect of bone grafting. However, concomitant procedures are frequent due to the presence of concomitant pathologies, which limits the generalizability of the current findings. The effect of concomitant bone grafting may be less in combined procedures. Finally, it should be noted that the present registry data are from more than 100 centres that are part of the German Cartilage Registry (KnorpelRegister DGOU). This heterogeneity of institutions and surgeons may influence the results. However, the results are more generalizable due to the multicentre design.

## CONCLUSION

The PRO and CRR in secondary M‐ACI is significantly higher at 36 months when patients are treated with additional ABG. High survival rates, comparable to primary M‐ACI, can be achieved with M‐ACI and ABG at 7 years. Subchondral bone should be addressed even in cases of mild bone involvement in revision M‐ACI. Further research is needed to identify patient‐specific and radiographic predictors in patients who may benefit from additional subchondral bone treatment.

## AUTHOR CONTRIBUTIONS

Johannes Weishorn performed the data extraction, statistical analysis and drafted the manuscript. Philipp Niemeyer and Thomas Tischer were involved in reviewing and drafting the manuscript. Peter Angele was involved in data acquisition and reviewing of the manuscript. Tobias Renkawitz reviewed the manuscript. Yannic Bangert served as supervisor of the study and was involved in reviewing and drafting the manuscript.

## CONFLICTS OF INTEREST STATEMENT

Philipp Niemeyer is an independent consultant for Arthrex, Stryker, Geistlich and Tetec. Peter Angele has received personal fees from Aesculap. Tobias Renkawitz declares the following conflicts of interest: Financial interests: Research funding at personal disposal: DePuy, Zimmer, Aesculap, German Federal Ministry of Education and Research, Deutsche Arthrose‐Hilfe, OttoBock‐Stiftung, German Federal Ministry of Economic and Development, Oskar‐Helene‐Heim Foundation in Berlin, Vielberth Foundation, Deutsche Forschungsgemeinschaft (DFG). Reimbursement of costs: DePuy, Zimmer, Aesculap, Federal Ministry of Education and Research, Deutsche Arthrose‐Hilfe, OttoBock‐Stiftung, Federal Ministry for Economic Co‐operation and Cooperation and Development, Oskar‐Helene‐Heim Foundation in Berlin, Vielberth Foundation, DGOOC, BVOU, DGOU. Reimbursement of costs for training/lectures: DePuy, Zimmer, Aesculap, German Society for Endoprosthetics (AE), Bavarian Association of General Practitioners. The other authors declare no conflict of interest.

## ETHICS STATEMENT

The current study was approved by the Ethics Commission of the Medical Center, University of Freiburg: EK‐FR 105/13_130795. Written informed consent was obtained from every patient before inclusion.

## Data Availability

The data will be available upon reasonable request.
